# A tool for theory development in action research: a challenge and a resource for nursing research

**DOI:** 10.1016/j.ijnsa.2025.100357

**Published:** 2025-05-28

**Authors:** Mary Casey, David Coghlan, Áine Carroll, Diarmuid Stokes

**Affiliations:** a UCD School of Nursing, Midwifery & Health Systems, University College Dublin, Dublin 4, Ireland; b Trinity Business School, University of Dublin, Trinity College, Dublin 2, Ireland; c School of Medicine, University College Dublin, Dublin 4, Ireland; d National Rehabilitation Hospital, Dun Laoghaire, Co Dublin, Ireland; e The Library, University College Dublin, Dublin 4, Ireland

**Keywords:** Action research, Quality, Quality criteria, QuARC checklist, Quality action research, Theory development

## Abstract

Action research is about action and research: both practice and theory. Theory development takes place throughout action research as the co-researchers strive to create meaning from their experiences of the change while the change is still occurring. Developing theory in action research may manifest in the creation of academic and practical knowledge. Failure to demonstrate the quality of action research reduces both its scientific value and practical application and so theory generation and development during the action research project maybe limited. Ensuring rigor in action research requires that the researcher is clear about the nature of the action or intervention, the ways in which data are collected and analysed and the processes through which the resulting theory is developed. Therefore, the activities and processes involved in using and generating theory in action research must be clearly reported through manifestation of the quality of the research.

This paper explores how a Quality Action Research Checklist (QuARC) tool provides a path to how ‘good theory’ may be generated through action research. It provides some background to the elements chosen for the QuARC and discusses key aspects established in the literature on the role of theory in action research. A second aim is to discuss the four areas in the QuARC tool and explore what action research projects should aspire to under these headings, and particularly how theory development may relate to these headings.

**Contribution of the paper**

**What is already known about the topic**

• Frequently action research is seen as atheortical

• There is a persistent failure to demonstrate both the scientific value and practical application of action research

**What this paper adds**

• Theory generation and development using an the action research approach will be enhanced through the application of the QuARC tool.

• Application of the QuARC tool ensures rigor in action research by establishing clarity about the nature of the action or intervention, the ways in which data are collected and analysed and the processes through which the resulting theory is developed.

Alt-text: Unlabelled box

## Introduction

Action research is a form of research where action researchers intervene in organizations and community systems to address real issues and through that action cogenerate practical knowledge in collaboration with those who are affected by the issues (Coghlan, 2023). As a science of action, it constitutes a different form of social science from often-used quantitative-qualitative categorizations with a distinct philosophy, epistemology, methodology and methods (Susman and Evered, 1978). In action research, theory and practice are inextricably entangled and some authors (Rolfe 1996) argue that one of its best uses is its narrowing of the theory-practice gap with claims to universality on the one hand and specific practice situations on the other. Ever since the seminal work of Archie Cochrane in 1972 on evidence-based medicine, an essential part of delivering health care is learning how to put theory and knowledge into practice and how to problematize and problem-solve the sometimes overwhelming issues that are all too common in under-resourced, struggling health service contexts. Action research is ideally suited for researching practice because of its situational, collaborative, participatory and self-evaluative nature. In addition, it has been identified as an appropriate method for investigating complex phenomena such as healthcare systems (Phelps and Hase, 2002; Biggs et al., 2021). Furthermore, it has the potential for developing theory that will be of relevance to practice because each intervention provides an opportunity for the researcher(s) to revisit theory in order to design the intervention, and to develop it further. To this point it is important to note how, while having process similarities to approaches such as project management and quality improvement, action research differentiates from these approaches by its essential emphasis on knowledge contributions beyond a local setting (Coghlan et al., 2023). To adapt a phrase from Kant, theory without practice is sterile and practice without theory is blind. The role of theory in action research is to deepen the understanding of practical challenges and to indicate what courses of action are open in each specific situation (Gustavsen 2008).

Many terms are used to describe action research approaches such that it is considered as a family of approaches (Author). According to Brydon-Miller et al., 2020 there are multiple forms of research that combine the principles of participation, action, and critical knowledge generation and that share a common commitment to using research to promote social justice. Indeed Durcikova et al. (2018, p. 243) suggest “there is no *a priori* reason, that existing forms of AR are the only legitimate forms of AR” and this gives rise to some of the added complexities of designing action research for theory development with due regard to the need to demonstrate methodological rigor. This means that, as there are future possibilities of development of different variations of action research, the establishment of criteria to establish the quality of action research in general is needed to avoid the future risk of criticism for being atheoretical and non-scientific. For instance, in an editorial for the journal. *Action Research*, Bradbury et al. (2025) present quality in terms of seven choicepoints, which combine objective, intersubjective and subjective element. Like all other types of research, ensuring rigor in action research necessitates that the researcher is clear about the nature of the action or intervention, the ways in which data are collected and analysed and the processes through which the resulting theory is developed. The aim of this paper is twofold. Firstly, to discuss key aspects established in the literature on the role of theory in action research. Secondly to explore how the four areas in the QuARC tool and what action research projects should aspire to under these headings, and particularly how theory development may relate to these headings. The paper does this through discussing some widely agreed principles of action research and relating them to the key themes and questions of the QuARC tool.

## Action research and theory development

As Varpio and Ellaway (2021, p. 341) observed, as much as theories are abstractions, they are “intimately and directly connected with practical experiences”. The activities and processes involved in using and generating theory in action research are essentially the book ends of the action research project which must be clearly demonstrated and reported in the methodology through manifest description of the quality of the research. Although there can be many different types of action research, they have a common feature namely that the researcher(s) take action and actually intervene in the context under study, working with key stakeholders and participants as co – researchers on matters of genuine concern to them (Huxham, 2003). Its outcome is a practical knowledge (Coghlan, 2016), or praxis (Eikeland, 2015; Kemmis 2006). Hence, in consideration of the myriad of types of action research, for the purpose of this paper, we chose a definition of action research that essentially underpins and captures the core elements of all forms of action research. Therefore, action research is defined as

… an emergent inquiry process in which applied behavioural science knowledge is integrated with existing organisational knowledge and applied to solve real organisational issues. It is simultaneously concerned with bringing about change in organisations and developing self-help competencies in organisational members and adding to scientific knowledge. Finally, it is an evolving process that is undertaken in a spirit of collaboration and co-inquiry” (Author) .

From this definition, it is clear that action research encompasses action on real issues (i.e. intervention in an existing practice in a particular social or healthcare context to bring about improvement and change) and research (i.e. systematic observation and analysis of the change that occurs as a result) by employing an initial recognizance or pre-step where the problem to be resolved is essentially mapped out, followed by iterative spiralling steps of (i) constructing, (ii) planning action, (iii) taking action, and (iv) evaluating action (Author). As highlighted, action research is emergent and real, it unfolds and can change during the process of the actual project in response to new understandings or identification of a new variable or theoretical explanation. Hence collecting data as it is happening during the action research process can provide both new and unexpected insights so that theory development processes are inductive leading to emergent theory. Moreover, strict adherence to a pre-determined data collection plan may not be possible in order to promote the emergence of new and creative insights. Very simply, because of the dynamic real-life nature of the project, the researcher(s) may not know in advance what or whom are the best sources of data to inform the study. In addition, the researcher(s) cannot always know in advance exactly what analysis process will be used, because the development of emergent theory requires the researcher to revisit the data with the co-researchers over prolonged periods of time in order to check out assumptions and reframe the data to generate new understanding which is all part of the action research cycle. Therefore, the methodology, as well as the theoretical and practical outputs, are almost always emergent because the researcher cannot know in advance what intervention opportunities will arise, or what past interventions may suddenly need to be revisited. These challenges make undertaking action research a complex process and can further complicate how rigor or quality are addressed in a study.

The object of action research is to generate theory that will be of practical value to practitioners as well as meeting the criteria for rigorous research. Hence action research intentionally merges theory with practice on the grounds that actionable knowledge results from the interplay of knowledge with action (Eden and Ackerman 2018). Actionable knowledge refers to knowledge that is both rigorous and relevant (Wood et al., 2023). We support Lewin’s argument that “…if the theorist does not look to applied problems with highbrow aversion or fear of social problems, and if the applied psychologist realizes that there is nothing so practical as a good theory (p. 288).” (Lewin, 1951 p 169), then this means that the theory must be expressed in such a way that it is possible to design practice as a reflection of the theory. However, action research transcends the mere concatenation of action and research. Hence, neither the action nor the research can be properly planned, conducted, or evaluated separately from the other. Rather it is the interaction of the action and the research and vice versa that constitutes action research and, in that sense, it is a more holistic research approach in that the whole is greater than the sum of its parts.

The word theory is derived from the Greek word for to ‘look or gaze’—that is what theory does. Theory provides a conceptual model for research, and research in turn contributes to theory. It has been defined as a big idea that organizes many other ideas. According to Doherty et al., 1993, p. 20 “theorizing is the process of systematically formulating and organizing ideas to understand a particular phenomenon. A theory is the set of interconnected ideas that emerge from this process”. According to Dick et al. (2009), theory is part of action and therefore of action research. When people act, they hope that their action will have the intended outcomes and if these are not achieved, usually people want some understanding or an explanation. Therefore, before people act, they have a theory, perhaps informal, connecting actions and results. They may think of it as ‘knowledge’ or ‘understanding’, which are other words for theory (Dick et al., 2009, p. 7), which helps to make sense of the event. He suggests that the term “theory is a grand word for these everyday activities of knowing, understanding and making sense”. Therefore, theory has a unique role in action research and there are two processes to consider, namely using theory and developing theory. Using theory enables action researcher(s) to collect rich data on what people do and say and what theories are used and useable to clarify and describe the dynamics of the issues that are of genuine concern to them. On the other hand, developing theory enables action researcher(s) to strengthen their processes of inquiry to offer possible explanations or hypotheses to create intended theoretical and practical outcomes. According to Dick et al. (2009) the former might be thought of as ‘content theory’, and the latter as ‘methodological theory’. In addition, Elden (1979) stressed the importance of developing local theory that is applicable to the local context in which the action research is occurring.

Using theory in action research maybe an inductive and/or deductive process and is manifest usually at the beginning of the research project when setting out the context of the study and examining the current literature on the state of knowledge about the topic. An inductive process is a bottom-up approach where the researcher and co researchers collaboratively collect and analyse data to develop theories, concepts or hypothesis based on the data and experience in practice. They usually start with specific observations and from these, they develop new understandings and then they move on to more general theories. Deductive reasoning draws on generalisable theory to craft particular arguments and provides explanation (Author) which is also known as top-down logic and is defined as the ability to make inferences about the veracity of a conclusion based on several, often competing, hypotheses. It involves logical reasoning whereby, if the theoretical or practical premises are true, then it follows that theoretical and practical conclusions must also be true. Deductive reasoning may draw on extant general theory to offer an explanation about the initial stages of teamwork development for example, on the project. Using extant theory based on the literature typically emerges from the diagnosis step and guides the action plans. Therefore, it may be seen that using theory is an earlier process in the action research project while generating theory may take place more towards the middle and latter end of the project when making sense of the data, generating outcomes, and engaging in discussion on how the current project adds to the existing theory and knowledge. Hence, within the action research project, theory grows from using extant theory, perhaps at the beginning, to theory development towards the end by moving from ‘just research’ to ‘practice-based research’, so that when brought together they enrich one another.

Using and generating theory in action research is manifest in the creation of academic and practical knowledge. As action researchers engage in first- and second-person inquiry and practices in the present tense in the pursuit of practical knowledge they also drawn on abductive reasoning (Author) as the co researchers cycle through the puzzles and anomalies that arise in the unfolding of the interventions. Achieving the final outcome(s) involves a good deal of abductive reasoning which offers plausible explanations about an issue or set of circumstances. While deduction draws on what ‘*must be’*, based on current knowledge and theory to craft an argument, induction can show or explain that something ‘*is operating’* in the present tense based on particular observations to clarify more generalisable theory, whereas abduction is more of a suggestive explanation of something that ‘*may be’* (Locke et al., 2008) and so it produces tentative answers and exploratory hypotheses in a move towards theory generation. While the term ‘research’ can be described as validating old knowledge and creating new knowledge and understanding, using theory and theory generation are necessary elements of action research in the creation of actionable knowledge. Thus, action research draws on the past, includes consideration of current actions and thinking with a view to taking action and offering a possible theoretical explanation of what may take place in the future. Linking these three knowledge development phases is just one aspect of the uniqueness of action research. In this way, theory provides the basis for evaluating the outcomes of the change or intervention. Knowledge in this sense is about the creation of facts, information, and skills on a topic acquired through experience or education. Research helps the creation of such knowledge and understanding. Academic knowledge refers to understanding an aspect of a phenomenon in an abstract way, while practical or practitioner knowledge relates to how people experience such a phenomenon, how they understand this experience and act upon it. Researchers demonstrate the results or outcomes of action research in terms of ideas, concepts, relationships, and frameworks. These might be seen as distinct knowledge components, but in some instances, these components may offer an explanatory statement and so consist of a tentative or embryonic theory development. In contrast, practitioner knowledge calls for the demonstration of the outcomes of action in the everyday language of participants. This may result in praxis where action research demonstrates how research can be useful and relevant for practitioners in the provision of knowledge of “what to do” as well as knowledge of “how to do”.

## The rationale for QuARC tool

Throughout a project, action researchers may consciously and deliberately move to and fro between academic knowledge and practical knowledge (Author) in order to recognize the value of both types of knowing in a given context. Hence activities of using and generating theory might be visible throughout the project as the researcher(s) strive to create meaning from their newly acquired knowledge, understanding and experiences of the problem issue, to endeavouring to solve the problem while the problem is still occurring. In this way, the process of theory generation culminates in deep learning over the course of the project. This contrasts with using extant theory in a literature review at the beginning of the research study and assuming it will reach definitive, generalisable conclusions. Therefore, action research both uses and generates theory through these processes and in this way, the culmination of knowledge creation or outcome(s) of the study results in practical and theoretical outcomes. These theoretical and practical outcomes, while occurring throughout the study, are usually located towards the latter end of the written project and manifest in the results, discussion, conclusion, and recommendation sections.

According to (Author) action research demands an explicit concern with theory that is generated from the conceptualization of the particular experience in ways that are intended to be meaningful to others. Yet action research is criticised for being subjective, anecdotal, unscientific, and not generalisable. Indeed, Levin (2003, p. 280) suggests that many see it as “offensive and illegitimate” and this directly relates to the lack of overt demonstration of scientific rigor or quality. This has culminated in a poor uptake of action research projects and a lower priority for publication, in view of an erroneous perception of its limited applicability to clinical practice. There is no doubt that a failure to demonstrate the quality of action research reduces both its scientific value and practical application and so the outcomes of the action research project in the form of theory generation and development maybe limited.

The establishment of rigor in action research is further hampered by a lack of a detailed checklist to provide operational guidance on how to proactively design ways to address and demonstrate quality in action research with a view to creating valid theory. This shortcoming makes it more difficult not only to design for theory development but also to balance the action and the research to successfully implement action research. In the light of previous reporting that many action research studies often lacked the specificity and details needed to communicate the context, quality of relationships, quality of the action research process itself or the dual outcomes, with enough precision, accuracy and thoroughness to allow readers to assess the design, execution of the work and the contribution theory a checklist was designed (Author). The purpose of the Quality Action Research Checklist (QuARC) tool was to demonstrate how good theory can be generated through the application of the quality criteria.

## The QuARC checklist

The Quality Action Research Checklist (QuARC) for reporting action research studies (Author) is based on a quality framework by (Author). It consists of four interrelated factors: context, quality of relationships, quality of the action research process itself and the dual outcomes of action and research. The aim of the checklist is to encourage researchers and practitioners to provide complete and transparent reporting on the quality criteria of action research, so that the outcome(s) of the research such as practical solutions and theory development are reliable and valid within the context and scope of the action research study itself. In this way, the application of the QuARC provides a framework for what ‘good’ use of theory in action research looks like.

## QuARC as a process of theory development

This paper poses the question as to how the Quality Action Research Checklist (QuARC) tool might provide a path to how ‘good theory’ may be generated through action research. We now discuss each factor

### Context

Articulating the practical and theoretical context of an action research initiative is foundational as it sets the scene for both the action and the research. With regard to the action process, both naming the concerns and forces for action – local, sectoral, national, global - and analysing the nature of these imperatives lays the foundations for the value of the work and anticipates the intended outcome. With regard to the researching process, uncovering both the stakeholders’ previous experience of the particular issue and how previous research sought to address the issues of concern and drawing on that experience and research to demonstrate understanding of the theoretical context of addressing the issues of concern provides a necessary parallel to framing the context of action. Such data capturing of the lived experience, would reveal the impact of the historical and/or cultural context on the participants and the use of theory via a review of the literature, would help offer explanation. In traditional research, this is often referred to as undertaking a literature review, though the lived experience and knowledge of the key stakeholders may not be readily captured.

In summary regarding context, the QuARC highlights the importance of searching for experienced participant accounts and extant theoretical explanations to promote understanding and to provide a foundation to add further knowledge and contribute to new theory development. It poses questions such as:

1. Is the action driven by a practical concern?
2. Is there local, national, and international imperative influencing the practical concern?
3. Does previous research inform the practical concern?
4. Does the action research project draw on previous research?

### Quality of relationships

Collaborative inquiry and action are the hallmark of action research, Hence the quality of the relationships involved in the process is paramount and impacts on theory development because it draws in and draws on all participants to have a shared understanding of what they all want to achieve or solve. Involvement of stakeholders throughout the project is essential in action research. In this instance, stakeholders are all participants within the scope of the research. Establishing good relationships between the researcher(s) and the participants is essential to bringing both perspectives of a particular issue or problem into view. In this sense, the quality of the relationship is emphasised to indicate that all participants are seen as co-researchers thus emphasising that no participant is portrayed as having ‘expert’ status be that from a research perspective or from a practitioner’s knowledge and experience. While all participants are taken to have equal status, the issue of power imbalance particularly where co-researchers include professionals and patients may result in relationships being of recognisably poor quality (e.g. patients/service users dropping out of the cycle or reducing their contributions). Capturing these challenges and how they are/are not resolved is essential to ensure that such conflicts are highlighted, so that such issues can be addressed in terms of the impact on theory development, rather than to simply 'judge' the quality of the AR on this basis. Hence, the outcome of the quality of relationships is a plurality of knowing which allows for the refinement of ideas and practice and lends itself to the creation of more robust theory from the process of enabling participants to question and challenge nascent ideas, process, and assumptions within a safe environment. Evidence of these interactions as well as reflective excerpts indicating how challenges were managed and overcome provide evidence on the quality of the relationships in the project.

The QuARC relates to the quality of the relationship of those engaged in the project in asking the following four questions: -

1. Are those who have articulated the practical concern in the first instance and who also have a stake its resolution included in all the steps of the action research cycle of constructing, planning action, taking action and evaluating?
2. Can the participants be classified as co-researchers?
3. Is the level of participation of the patients/clients and co-researchers evident at each step of the action research cycle?
4. Is the action research project evaluated in terms of the quality of the relationships?

### Quality of the action research process

Action research comprises cycles of action and reflection which constitute not only insights from the particular set of interventions taking place within the cycle but also through reflections on interventions or literature exploration gained from research cycles’ impact and influencing developments made in earlier cycles (Eden and Ackermann 2018). The quality of the action research comes from the ‘to-ing and fro-ing’ or the movement between the steps in the action research spiral (that is, constructing, planning, taking action, and evaluation) and the questioning of each. This dialectical process of action research which involves theory, research and practice increases understanding and abstraction of ideas alongside and between improvements in the real world. Practical issues in action research are typically addressed through the action research cycles in which the outcomes of each cycle are checked against plans and intentions—this is the essential evidence base of the research. As people engage in the action processes, periodically they are invited to reflect and evaluate the research procedures they are using as part of the actual research cycle. In doing so they clarify and extend their understanding of their research practices that inform their immediate quest, which may result in a change of direction but should provide useful, more general understandings (theory) about the dynamics of action research. Tentative hypothesis may be created and tested and evaluated in practice and researchers and practitioners are encouraged to draw on both theoretical and practice-based literature to help explain ongoing developments. Theory in this instance may relate to the particular issue investigated and to the research process in action. This dialectical research process between topic and method helps to build on existing knowledge and experience leading to theory refinement or indeed to new theory development.

The action research cycle develops theory in practice through an inquiry or operationalizion of an idea within a specific research methodology. The action part requires application of the research approach and reflection enables understanding of the use and impact of the approach and finally, as the researchers collaboratively look for theory to explain outcomes, they further develop the theory. Collaborative engagement on an equal status is essential from the initial stages of identifying or diagnosing the problem, to planning and executing action to finally evaluating that action and revisiting the issue again and beginning a new cycle. While the action research process obviously requires a minimum of one complete cycle to demonstrate each step, action researchers typically are not satisfied with one turn of the action research spiral but realise the necessity of repeating this process perhaps several times. Engagement in several cycles enables the co-researchers to check out underlying assumptions, different perspectives, and possible solutions on the topic of interest. The collaborative approach of action research seeks to overcome challenges to understand the ‘real picture’ through checking the congruence of others’ interpretations with the researcher’s own interpretation. This will lead to new understanding and synthesis of ideas and even perhaps to theory refinement or to new theory generation, which can then be tested for workability in the next action research cycle and so on. One of the findings from previous work was that the level of participation of stakeholders faded after the initial stages of the study got underway (Author) and in many cases, their involvement was not at all evident in any data analysis or evaluation activities. Hence, the quality of the action research cycles can be poor and theory development limited by a reduction in the opportunities for participation and shared understanding and concomitant theory refinement. Reports of evidence of engagement of the coresearchers would therefore reveal the complexities of people's situations as they attempted to change them and to QuARC poses such questions as.: -

1. Does the final account demonstrate a rigorous and collaborative engagement in the action research project’s design?
2. Does the account demonstrate subsequent enactment of cycles of constructing, planning action, taking action and evaluating?
3. Is enactment of the cycle towards the practical and theoretical objectives transparent and reliable?
4. Is the engagement of the coresearchers evident in shared data analysis? and shared

### Outcomes

Action research seeks to produce two outcomes – a practical outcome of the action and a contribution to knowledge for an audience beyond those who were directly involved. The quality of an action research project ought not be judged by its ability to effect change; the degree of change is not the measure of quality as indeed to decide not to take action might be a moral imperative in certain circumstances. Therefore, a report should indicate some analytical discussions on the process of change i.e. the outcomes of the action research and the difficulties encountered when attempting to act politically and ethically. The collaborative engagement by the co-researchers in shared reflection is an important activity in terms of outcomes and reporting on reflexivity of how the researchers’ own actions, beliefs and values have affected or are affected by the situation, and its interpretation is essential. Such reflexivity helps reveal meaningful changes to practice and to life circumstances. Therefore, engagement in reflection helps establish rigour by ensuring the reliability of the study as it helps to overcome the problem of introspection through unchallenged reflection that is not linked to theory.

QuARC poses such questions as asks the following questions: -

1. Are both forms of outcomes presented: theory (research) and action (practical)?
2. Are the outcomes sustainable?
3. Are the outcomes useful for other action research projects?
4. Does the theoretical outcome contribute to future theory development?
5. Is there a reflection on the process of engagement on the cycles by the coresearchers?

## Discussion

In this article we have posed the question, how might the QuARC (Quality of Action Research Checklist) tool contribute to theory development in action research? While theory development tends to be considered only as an outcome to research, we have taken a broader view and explored how all the quality factors of an action research initiative contribute to theory development. By discussing the four factors that make up QuARC, we have explored how the generation of theory and practical knowledge, the manifestation of the activities and processes of using and generation of theory in action research is essentially about demonstrating not only how each of the four criteria of the QuARC are addressed but also how they are linked. Indeed as it is the systemic connectedness of each of the four factors with each other that can constitute quality in action research, so it is through the linkages between all four quality criteria that theory is developed.

Demonstrating how each of the four criteria is addressed as well as providing a clear manifestation of the linkages between all four quality criteria in action research enables the process of using and generating theory in action research to be transparent and rigorous. Theory development in action research becomes known not only through its execution of scientific method by demonstrating how it upheld quality, but also through its contribution to the success or failure of the action. The ability of a theory to adequately capture the reality of a situation can be tested only through acting on it and seeing what happens. The success or failure of the action in action research becomes known not only through its impact on the problem experienced by the people in the field, but also through its contribution to increased understanding of the problem and in particular to practical and theoretical developments. Within an action research project, theoretical and practical insights are gleaned from (i) the process of understanding theory from the perspective of explanation and application in practice, (ii) the process of designing theory for practice so as to offer possible explanations, (iii) application of the theory in practice so as to get value from it and act on its implications, and (iv) undertaking further cycles of elaboration and redesign of ideas and theory(s) based on evaluation and its subsequent re-application. Action research therefore both uses and generates theory. An effective action research design will not only address the initial research questions or practical concern but will also yield valid data and offer possible explanations and ensure coherence between data collection, analysis and the final conclusions drawn while also ensuring that the theoretical and practical outcomes of a study are acceptable, dependable, and consistent. The QuARC enables the key elements of the action research design, and the conclusions drawn from it, to be broadly evaluated against these four quality criteria. The quality of the theory development in action research is dependent on addressing these criteria and successfully overcoming issues of quality within the research and developing theory. According to Gustavsen (2008, p.434) “the ultimate test of the fruitfulness of a theory is its ability to reach out in society, mobilise people and give rise to new practices”.

A complete and transparent explanation which manifests in adherence to quality criteria for action research must be provided in the final report. This enables current and future practitioners and action researchers to place value on the quality of the study and hence its theoretical and practical outcomes. In addition, researchers and practitioners must ask, how do we know that the action research outcomes or substantive theory are valid and have currency and not simply about “just telling a good story” (Author). Application of the QuARC checklist will facilitate greater adherence to establishing the quality of the action research project and therefore contribute to more reliable and valid theory development. However, it is also important to state that quality is less about adherence to these specific QuARC criteria than it is about demonstrating how theory is used and generated in action research. As described previously, in action research there is a kinship between the action component(s) and the scientific research. Accordingly, any discussion on quality must occur in the context of both of these aspects. Hence, using and generating theory is *sine qua non* in action research. This paper promotes the advancement of action research, by demonstrating that the application of the QuARC proactively can enhance the overall quality of the research and so enabling its contribution to both academic and practical worlds to be recognised through its contribution to activities of using and generating theory.

## Conclusion

This paper has demonstrated that the QuARC (Quality of Action Research Checklist) tool can play a crucial role in advancing theory development within action research. By emphasising the interconnectedness of the four quality criteria, generation of theory and practical knowledge, manifestation of activities, transparency, and rigor, the QuARC can ensure a comprehensive and systematic approach to action research. Through its application, researchers will be able to transparently address how theory is used and generated in practice, linking these elements to both the scientific and practical aspects of the research. This process not only enhances the reliability and validity of theory development but also fosters a deeper understanding of real-world problems. As action research inherently uses and generates theory, the ability to evaluate and refine theory through the QuARC checklist enhances the impact of research outcomes, ensuring they contribute meaningfully to both academic knowledge and practical applications. Ultimately, the QuARC tool can support the advancement of action research by promoting quality and facilitating the effective mobilisation of theory in society, as emphasised by Gustavsen (2008). Through rigorous adherence to these criteria, action researchers can ensure that their work is both valuable and impactful, offering insights that extend beyond the immediate research context and into new practices and societal change.

## Funding

The author(s) received no financial support for the research, authorship, and/or publication of this article.

## CRediT authorship contribution statement

**Mary Casey:** Writing – review & editing, Writing – original draft, Conceptualization. **David Coghlan:** Writing – review & editing, Conceptualization. **Áine Carroll:** Writing – review & editing, Conceptualization. **Diarmuid Stokes:** Writing – original draft, Conceptualization.

## Declaration of competing interest

The author(s) declared no potential conflicts of interest with respect to the research, authorship, and/or publication of this article.
